# Airborne fungi spores distribution in various locations in Lagos, Nigeria

**DOI:** 10.1007/s10661-019-8038-3

**Published:** 2020-01-03

**Authors:** Adeyinka Odebode, Adedotun Adekunle, Jason Stajich, Peter Adeonipekun

**Affiliations:** 1 Department of Botany, Faculty of Science, University of Lagos, Akoka, Nigeria; 2 Department of Environment and Natural Science, Kabale University, Kabale, Uganda; 3 Department of Microbiology and Plant Pathology, University of California, Riverside, USA

**Keywords:** Fungi, Airborne, Lagos

## Abstract

Exposure to outside air microorganisms especially fungi has been linked with illness such as allergic respiratory symptoms, rhinitis, asthma, and infection such as mycosis. Airborne fungal composition was sampled from five locations in Lagos State, Nigeria, between May 2014 and April 2016. Fungi spores were collected using the sedimentation plate method with the Petri dishes of dichloran-glycerol 18 (DG-18) and potato dextrose agar (PDA) media. Fungi sporulated faster on DG-18 agar plate as compared with PDA. The abundances of fungal spores collected monthly at the locations varied. The most abundant spores came from the fungi were *Aspergillus niger* (14.47%), *Aspergillus sydowii* (10.37%), and *Aspergillus flavus* (7.93%). Additional species were present in the collections including Ascomycetes: *Penicillium funiculosum* (5.49%), *Neurospora crassa* (5.32%), *Penicillium oxalicum* (4.71%), *Penicillium pinophilum* (2.88%), *Fusarium verticillioides* (3.05%), *Penicillium simplicissimum* (1.83%), *Aphaderanum* sp. (0.22%), *Curvularia* sp. (0.22%), *Aspergillus oryzae* (0.22%), and *Paecilomyces* sp. (0.61%) and the Mucoromycotina Zygomycetes: *Rhizopus oryzae* (4.10%) and *Mucor* sp. (3.44%). Fungal concentrations were significantly higher (*P* ≤ 0.05) during the rainy season compared with the dry season. *Aspergillus* and *Penicillium* were the most predominant airborne fungal genera while *Mucor*, *Alternaria*, and *Cladosporium* were some of the least observed. Generally, abundance of fungi was significantly high during the wet season in all the studied locations.

## Introduction

Airborne spore dispersal is an important reproductive dispersal mechanism for many genera of fungi. The small size and hydrophobicity of spores enable long-distance delivery of fungi and can have a great influence on human health and also on plant health. A large fraction of airborne spores originate from agricultural and outdoor environment (Odebode 2017) and the distance they travel depends on so many factors. Microorganisms are abundant in the atmosphere, but their distribution and abundance vary with prevailing environmental situation and also with locations. Usually, a greater concentration is found in urban areas than in the rural areas (Abdel Hameed et al. 2009). The density of airborne microorganisms displays topological, location effect, and seasonal differences (Ianovici 2008). As such, airborne microorganism abundance varies with time, year, and location (Bowers et al. 2013). Changes in humidity can also affect the abundance of fungal spores. The distribution of air microbes varies throughout the period in a day depending on weather parameters and has been shown to be influenced by environmental factors including type of vegetation around (Pepeljnjak and Šegvić 2003), air pollution (Lin and Li 2000a, 2000b), human activities going on (Mitakakis et al. 2001), meteorological parameters, and also seasonal variations (Rossi et al. 2005; Klaric and Pepeljnjak 2006). Airborne microorganisms have also been associated with spoilage of food (Tournas and Katsoudas 2005) and damage of books stored in libraries and also materials stored in archives (Aira et al. 2007). They have also been implicated in spread of plant and animal diseases (Rossi et al. 2005). Air distribution of bacteria and fungi poses significant health problems (Balasubramanian et al. 2011). Contact with airborne microorganisms has been linked with allergic respiratory symptoms, asthma exacerbation, asthma-related death, and infection (Dales et al. 2004a, b; Peternel et al. 2004). In Nigeria, little attention has been given to the study of airborne microorganisms. This study therefore investigates the composition and distribution of airborne fungi sampled across multiple locations for 2 years in Lagos State, Nigeria.

## Materials and methods

**Fig. 1:**
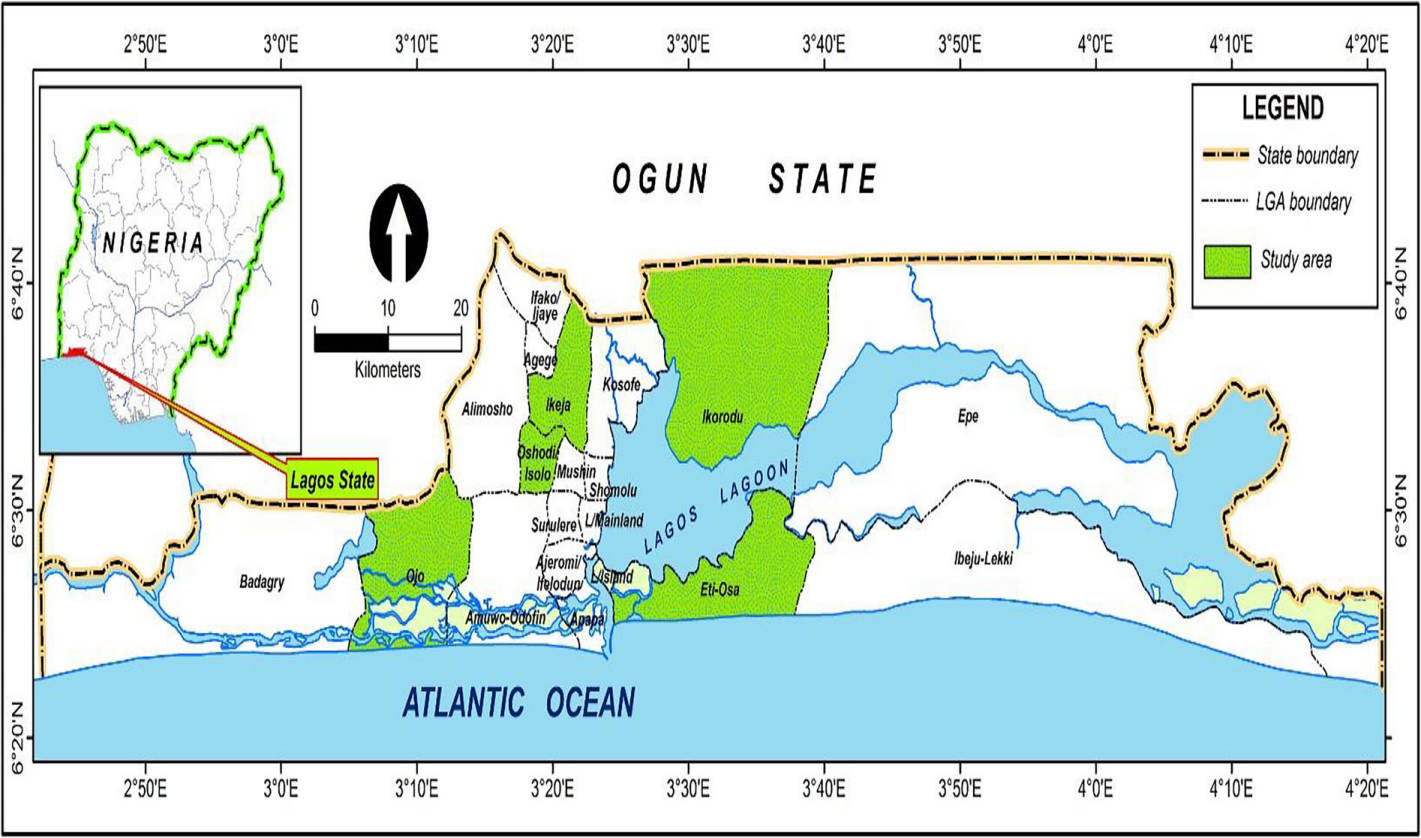
Map showing sampling locations of air spores from different locations in Lagos State, Nigeria

Five different local governments (county) spread across various parts of Lagos State, Nigeria, were selected for the study (Fig. 1). Except for Victoria Island and Oshodi, all the locations where sampling was done had some form of vegetation like few shrubs at a distance from the sampling location. The locations were chosen to examine a variety of human activities which include an outdoor market, a hospital, a residential area, a high school, and a commercial area.

### Atmospheric fungi collection

Airborne fungal spores were sampled monthly for the period of 24 months between May 2014 and April 2016 at five locations in Lagos, Nigeria (Table 1). The open plate method was used for sampling by opening sterile plates containing dichloran-glycerol 18 (DG-18) agar and potato dextrose agar (PDA). Plates were opened for 10 min at human height (1 m above feet level which is approximately the human breathing zone) and then recovered. Samples were collected in triplicates and transferred to the Mycology Laboratory of the Department of Botany, University of Lagos, and incubated at room temperature (28–31 °C) for 3 to 5 days. Colony count and growth appearance were monitored.

**Table 1:** Coordinate descriptions of different sampling locations

Location	Description	GPS
Iba	A farm settlement where fruits and vegetables are grown.	N6027′38, E3012′20
Oshodi	A densely populated area which serves as motor park, garage, and filing station with few buildings around.	N6033′18, E3020′75
Ikorodu	A cosmopolitan area with diverse activities including trading, motor park, housing, and bus stop.	N6038′51, E3025′25
Ikeja	A hospital environment where patients are attended to.	N6060′18, E3035′15
Victoria Island open recreational space with nearby residential buildings.		N6042′81, E3042′19

The same location was sampled monthly for the entire study period and was always visited at first week of every month.

### Identification of fungi

After detection of fungal growth on plates, the topography, texture, and pigmentation of each specific type of colony are noted in order to identify the fungi accurately. The identities of these fungi were identified using cultural and morphological characteristics and by also comparing them with confirmed representatives of different species in relevant texts such as Alexoupolous et al. (2007), Barnett and Hunter (1999), and Ellis (1971).

The percent frequency at which each fungus was observed was calculated as the number of the observations divided by the total number of colonies of fungi from all the sites, throughout the study months.

Molecular methods were also employed to support identification of the sampled fungi to overcome limitations of morphological identification using specific primers (Table 2).

**Table 2:** Primers used for fungi amplification

Locus	Primer name	Direction	Sequence	Target region
Internal transcribed spacer (ITS)	ITS 1	Forward	5′TCCGTAGGTGAACCTGCGG3′	18S rDNA
	ITS 4	Reverse	5′TCCTCCGCTTATTGATATGC3′	
Large ribosomal unit	LRO5	Forward	5′TCCTGAGGGAAACTTCG3′	LSU
	LROR	Reverse	5′ACCCGCTGAACTTAAGC 3′	

### DNA extraction

For DNA isolation, the cultures were grown in potato dextrose agar (PDA) for 7 days at 28 ± 1 °C.

Extraction of fungal DNA was done using modified cetyltrimethylammonium bromide (CTAB) protocol (Lee et al. 1998).

DNA extraction and polymerase chain reaction (PCR) were employed to amplify copies of the partial internal transcribed spacer (ITS) fragment of rDNA in vitro. The quality of the PCR product was assessed by undertaking gel electrophoresis. PCR purification step was carried out to remove unutilized dNTPs, primers, polymerase, and other PCR compounds in order to obtain a highly purified DNA template for sequencing. This procedure also allowed concentration of low-yield amplicons.

### PCR amplification program

94 °C for 5 min, 40 cycles of 94 °C for 45 s, 48 °C for ITS1 (52 °C for TEF and ACT) for 30 s, 72 °C for 90 s and a final step at 72 °C for 6 min. All amplicons were sequenced as described by Carbone and Kohn (1999).

### Quantification of isolates

Spectrophotometry was used to quantify the DNA obtained and purity was also determined from the A260/A280 ratio averaged (> 1.77).

The amplified PCR products were electrophoresed on a 1% agarose gel in TBE buffer visualized by staining with ethidium bromide and photographed using a gel documentation system.

### Gel extraction and sequencing

Gel extraction and Sanger sequencing of amplicon products were performed using ABI 3730 (Institute for Integrative Genomes Biology, Genomics Core facility, University of California, Riverside). After sequencing, the sequences obtained were blasted using Basic Local Alignment Search Tool (BLAST) to identify the closest affiliated sequences in NCBI database.

### Statistical analysis

Fungal spores data obtained were analyzed using multiple analysis of variance (ANOVA) and means were separated using Duncan multiple range test (DMRT) with the level of significance at *P* < 0.05 (95% confidence interval). Histograms and line graphs were also used for graphical representations.

Rainfall data was obtained from Nigerian Meteorological Agency, Oshodi, Lagos, Nigeria.

## Results

Below are the results of the 23S rRNA data of the sequenced fungi (Table 3).

**Table 3:** Molecular identification of fungi isolated from different sampling sites

Sample	Organism	Match identity (%)	*E* value	Query cover
31 ITS	*Aspergillus aculeatinus*	99	1e	285
1 ITS	*A*. *flavus*	94	3e	345
25 ITS	*A*. *fumigatus*	97	3e	305
24LR	*A*. *niger*	99	1e	138
34LR	*A*. *niger*	95	2e	301
6LR	*A*. *ochraceus*	98	3e	175
9 ITS	*A*. *oryzae*	99	2e	302
41 ITS	*A*. *oryzae*	96	2e	233
30 ITS	*A*. *penicilloides*	96	3e	175
14LR	*A*. *protuberus*	97	4e	309
4LR	*A*. *subramanianii*	99	3e	169
29 ITS	*A*. *tamari*	96	3e	238
5LR	*A*. *tubingensis*	93	4e	254
36 ITS	*Absidia blakesiana*	96	1e	197
39 ITS	*Aphaderanum* spp.	97	2e	192
7LR	*Aspergillus terreus*	95	2e	256
19 ITS	*Cladosporium herbarium*	97	3e	201
14 ITS	*Curvularia* spp.	95	2e	145
10 ITS	*F*. *verticillioides*	99	4e	269
17LR	*Fusarium sublunatum*	99	4e	239
13 ITS	*Mucor* spp.	96	3e	166
35 ITS	*Neurospora crassa*	97	4e	234
40 ITS	*P*. *chrysogenum*	98	3e	256
16LR	*P*. *citrinum*	86	3e	345
20LR	*P*. *citrinum*	97	1e	198
25LR	*P*. *oxalicum*	99	3e	209
26LR	*P*. *pinophilum*	96	1e	354
27LR	*P*. *simplicissimum*	98	2e	233
33LR	*Paecilomyces* spp.	99	1e	276
11LR	*Penicillium funiculosum*	100	2e	147
23LR	*Perenniporia koreana*	97	2e	324
33 ITS	*Phoma eupyrema*	98	1e	304
12 ITS	*Rhizopus stolonifer*	97	2e	197
21 ITS	*Sistotrema brinkmanii*	95	1e	179
19LR	*T*. *asperellum*	97	3e	198
8LR	*T*. *harzianum*	87	2e	321
7 ITS	*T*. *helicum*	97	2e	230
32LR	*T*. *viride*	99	3e	124
18LR	*Trichoderma harzanium*	98	3e	234

**Fig. 2:**
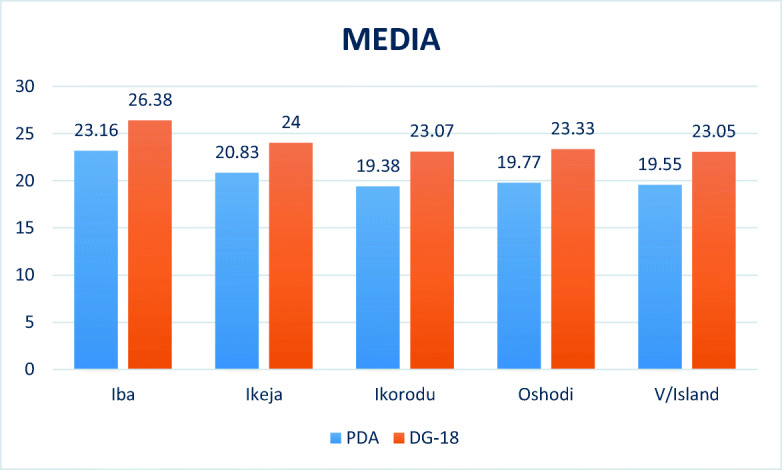
Abundance of fungal spores in different locations in Lagos State

Means with different superscripts are significantly different. Mean separation was done with the Duncan multiple range test at *P* < 0.05. Abundance of fungal spores in different locations with respect to media showed that for the different locations in Lagos State, Iba produced significant difference (*P* < 0.05) from other locations for both PDA and DG-18 agar followed by Ikeja (20.83 and 24.00) while Ikorodu had the lowest value (19.38) with respect to both media (Fig. 2). In all the locations, DG-18 media produced significant difference than PDA (Table 4).

**Table 4:** Monthly comparison of abundance of fungal spores isolated

Year	Months	PDA	DG-18
2014	May	23.40cd	27.20bc
	June	25.20ab	29.20b
	July	23.40cd	26.40cde
	August	21.20fg	24.40ef
	September	21.20fg	24.80def
	October	21.60efg	24.20f
	November	17.20h	20.00ghi
	December	14.80i	18.40i
2015	January	17.40h	21.20g
	February	17.20h	20.80g
	March	20.00g	23.80f
	April	21.40fg	24.40ef
	May	23.20cde	26.80cd
	June	26.00a	31.40a
	July	24.00bc	27.20bc
	August	21.80def	25.00def
	September	22.00def	25.60cdef
	October	22.00def	24.60ef
	November	17.40h	20.20ghi
	December	15.20i	18.60hi
2016	January	17.40h	21.60g
	February	17.20h	20.60gh
	March	20.60fg	24.00f
	April	21.80def	23.80f
	LSD	1.65	2.05
	EMS	1.74	2.66

The second year of collection had higher monthly percentage fungi spores collected than the first year. There was gradual increase in fungal spore collection from the months of March, April, and May, and highest collection was in June. Higher values were observed during the periods of May–August which are significantly different (*P* ≤ 0.05) from the values observed early and later part of the year (Table 4).

**Fig. 3:**
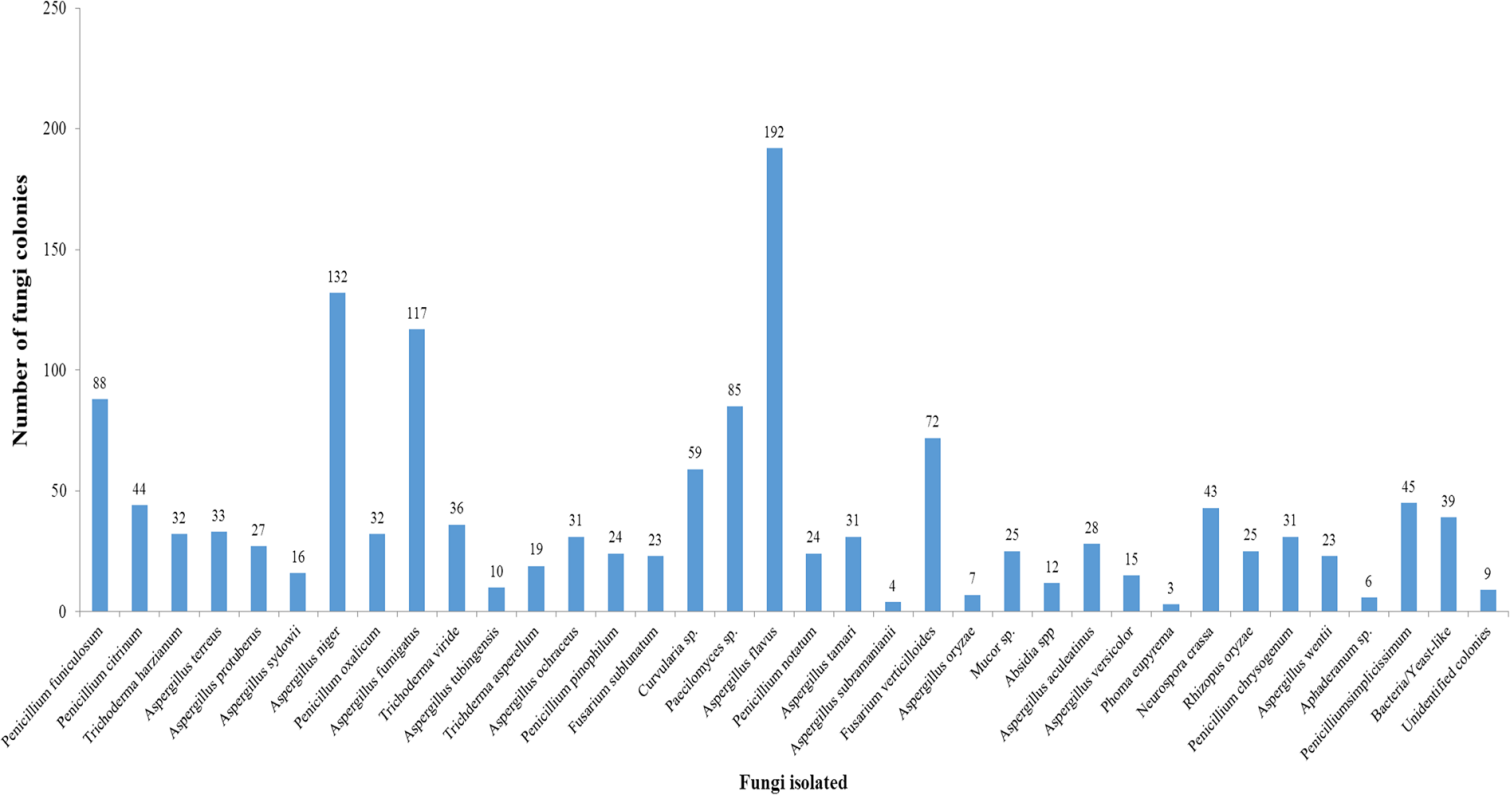
Abundance of fungi isolated in Iba, Lagos state

Thirty-six different fungal spore types were collected from Iba. The atmosphere was dominated by fungal spores from majorly Ascomycetes and Deuteromycetes. *Aspergillus flavus* was the most frequently collected followed by *Aspergillus niger* and *Penicillium funiculosum*. The fewest spores were recorded from *Phoma eupyrema*, *Aspergillus subramanianii*, and *Aphaderanum* sp. Other fungal species include *Penicillium funiculosum*, *Aspergillus protuberus*, *Fusarium verticillioides*, *Neurospora crassa*, and *Penicillium citrinum* (Fig. 3).

**Fig. 4:**
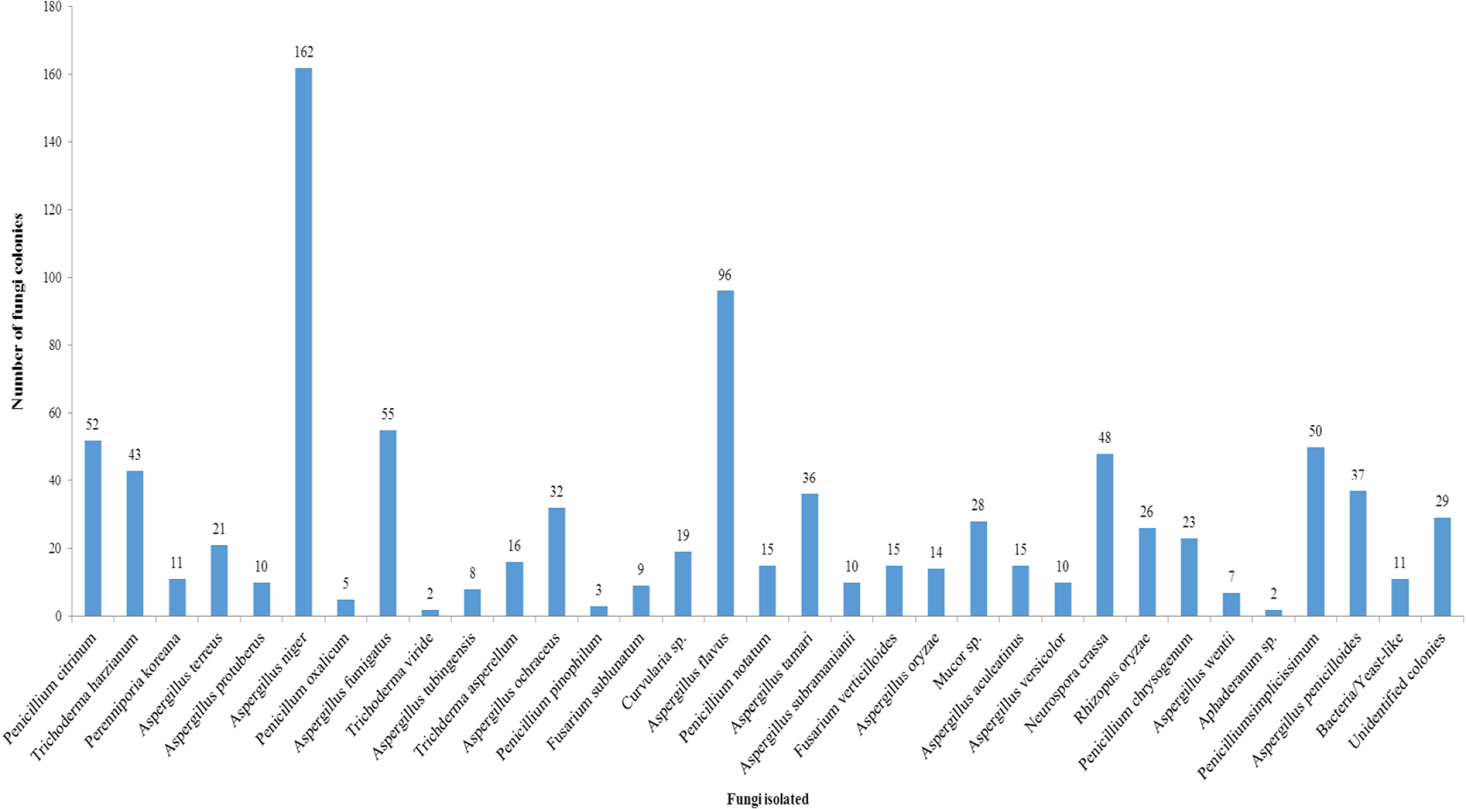
Abundance of fungi isolated in Ikorodu, Lagos State

Thirty-four fungal spores belonging to different species were isolated from Ikorodu location. The atmosphere was dominated with varied fungal species of different fungal spores especially those of Ascomycetes and Deuteromycetes including *Aspergillus niger*, *A*. *flavus*, and *A*. *fumigatus*. Lowest record of fungal spores was observed for *Aphaderanum* sp., *Trichoderma viride*, and *Penicillium pinophilum* (Fig. 4).

**Fig. 5:**
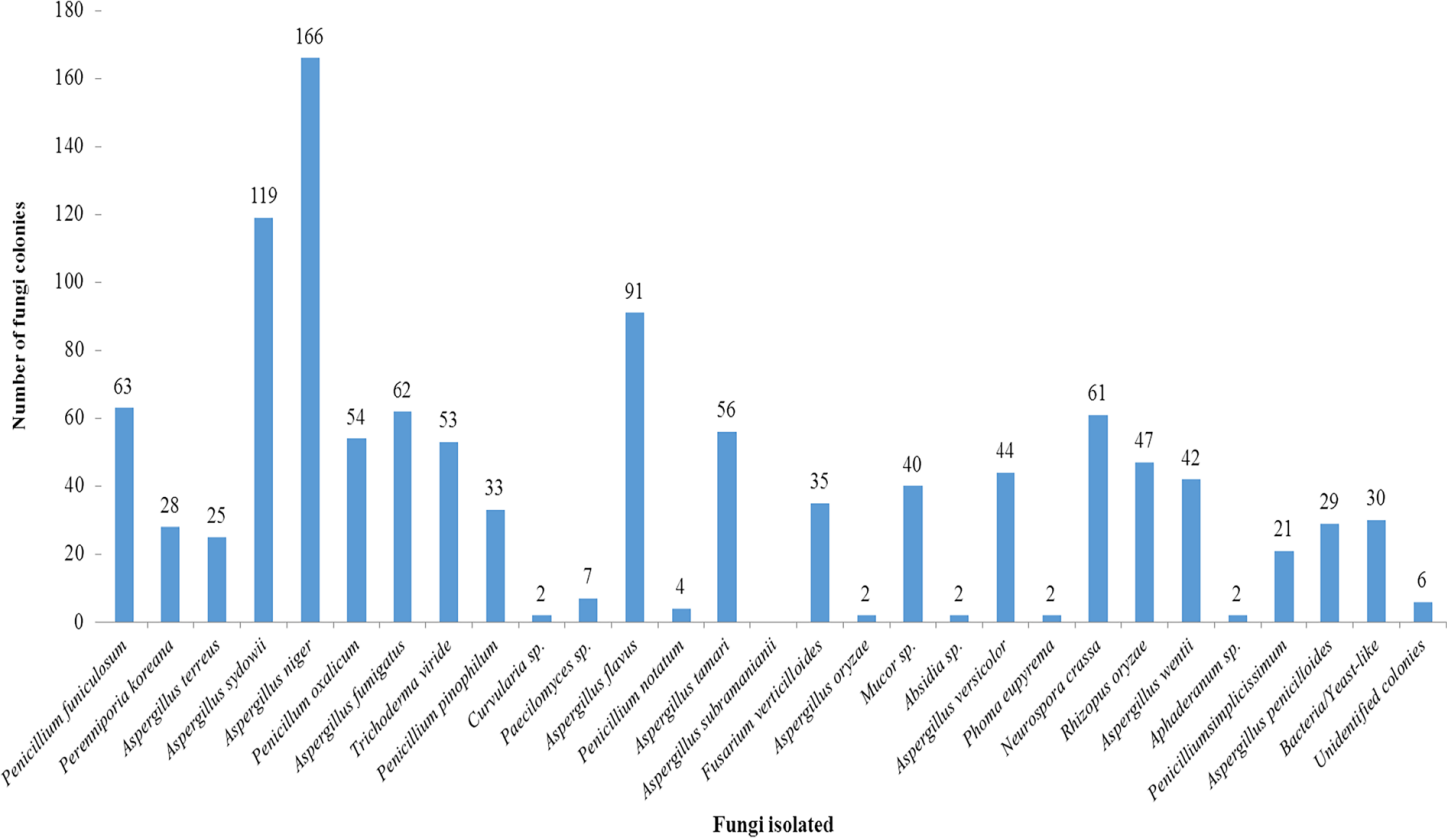
Abundance of fungi isolated in Ikeja, Lagos State

Twenty-eight fungal spores were recorded from the aeroflora for the duration of sampling belonging to different families for Ikeja location. Dominant spores were *A*. *niger*, *A*. *sydowii*, and *A*. *flavus*. Others which were sporadic include those of *Aphaderanum* sp., *Curvularia* sp., and *A*. *oryzae*. Other fungi spores isolated also include *Paecilomyces* sp., *Mucor* sp., *Neurospora crassa*, *P*. *funiculosum*, *P*. *simplicissimum*, *P*. *oxalicum*, *P*. *pinophilum*, *F*. *verticillioides*, and *Rhizopus oryzae* (Fig. 5).

**Fig. 6:**
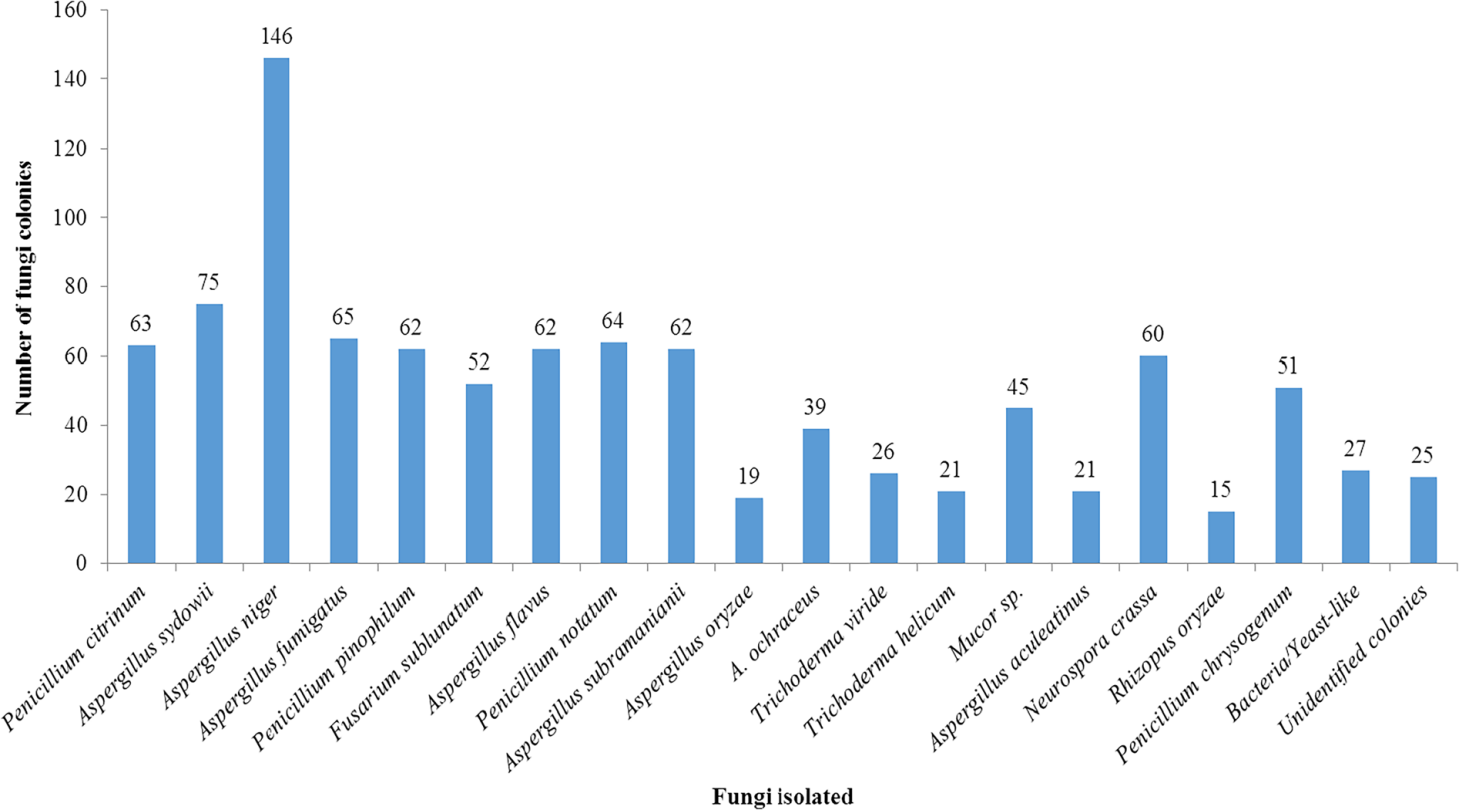
Abundance of fungi isolated in Victoria Island, Lagos State

Twenty fungal spores were identified from aeroflora of Victoria Island, Lagos, Nigeria. Dominant fungi include those of *Aspergillus niger*, *A*. *fumigatus*, and *Penicillium notatum*, while spores of *Rhizopus* sp., *T*. *helicum*, and *A*. *oryzae* recorded the lowest abundance of spores. Other spore types were identified, among which include *A*. *aculeatinus*, *P*. *pinophilum*, *P*. *citrinum*, *Fusarium sublunatum*, *Trichoderma viride*, and *Mucor* sp. (Fig. 6).

**Fig. 7:**
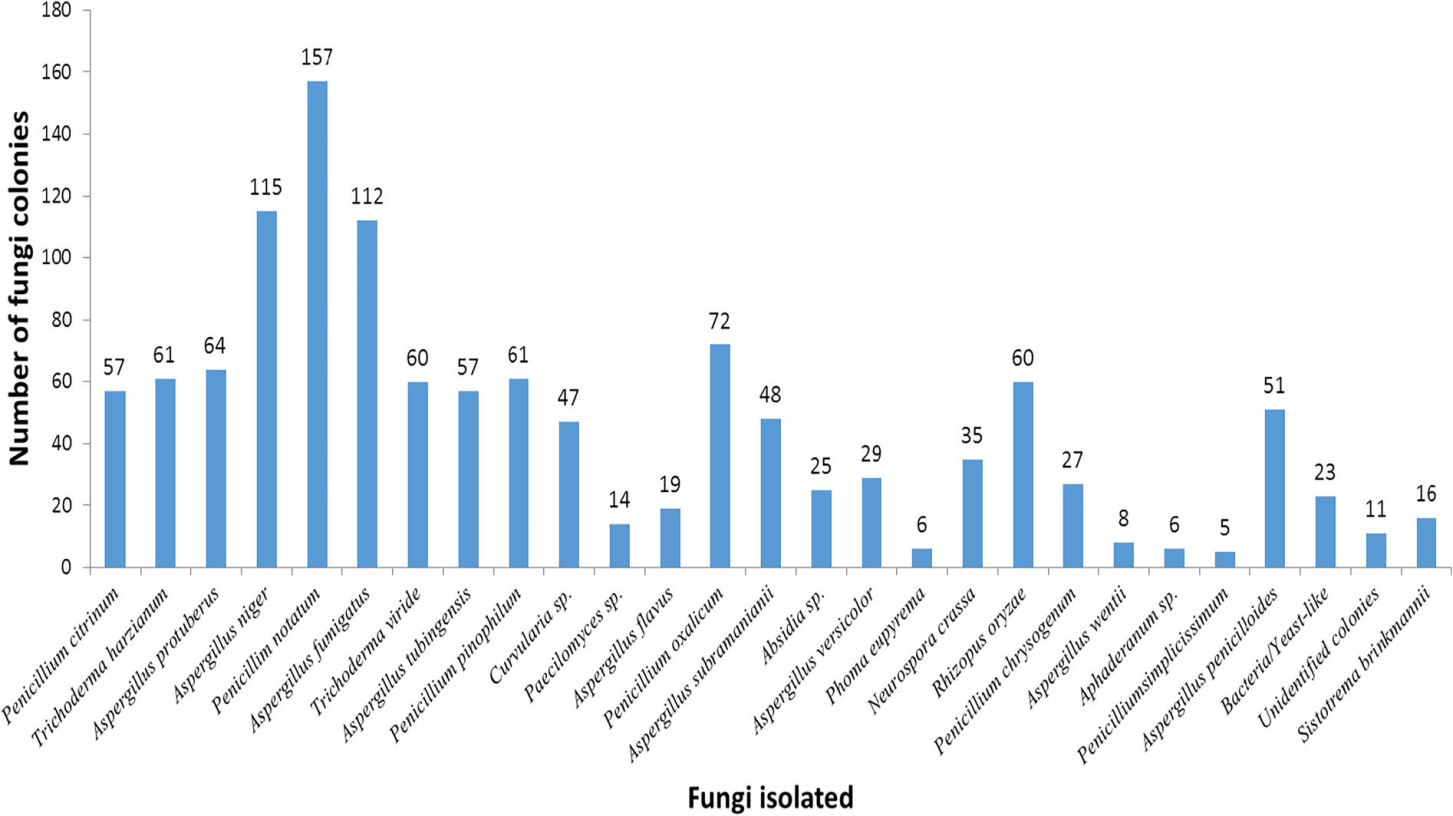
Abundance of fungi isolated in Oshodi, Lagos State

**Fig. 8:**
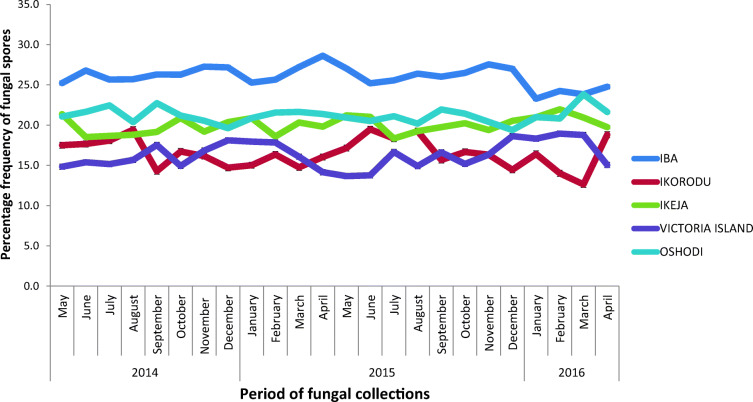
Frequency of fungi occurrence in Lagos, State

Twenty-seven different fungal spores were isolated from Oshodi location. Ascomycetes and Deuteromycetes spores were the major contributors. *Penicillium notatum*, *A*. *niger*, and *A*. *fumigatus* were the most occurring fungal spores while *P*. *simplicissimum*, *Aphaderanum* sp., and *Phoma eupyrema* were the lowest in abundance (Fig. 7). The frequency chart also showed that Iba had the highest fungal spore collection during the 2-year survey (Fig. 8).

**Fig. 9:**
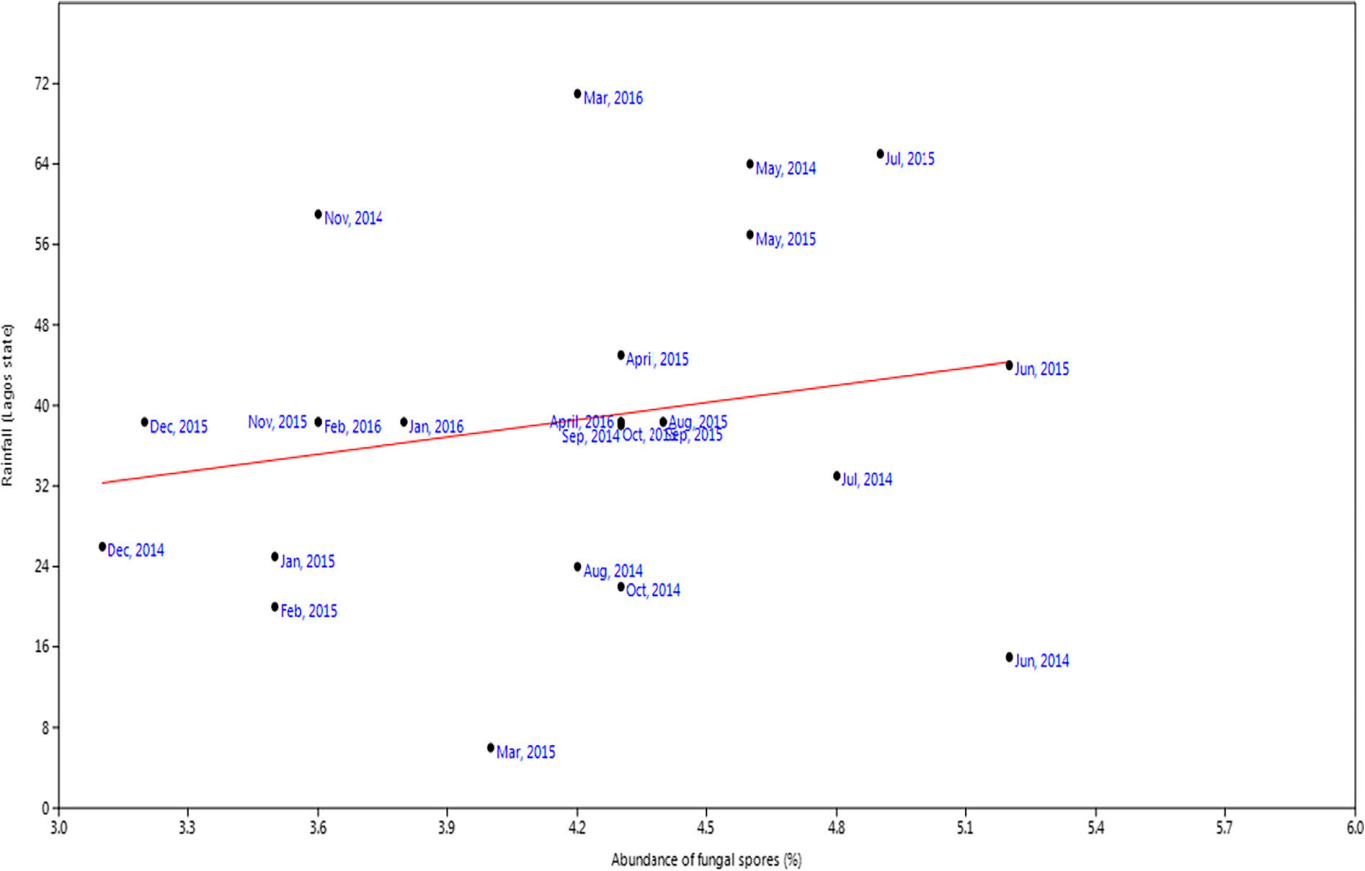
Multivariate linear regression between fungal spore abundance and rainfall in Lagos

**Fig. 10:**
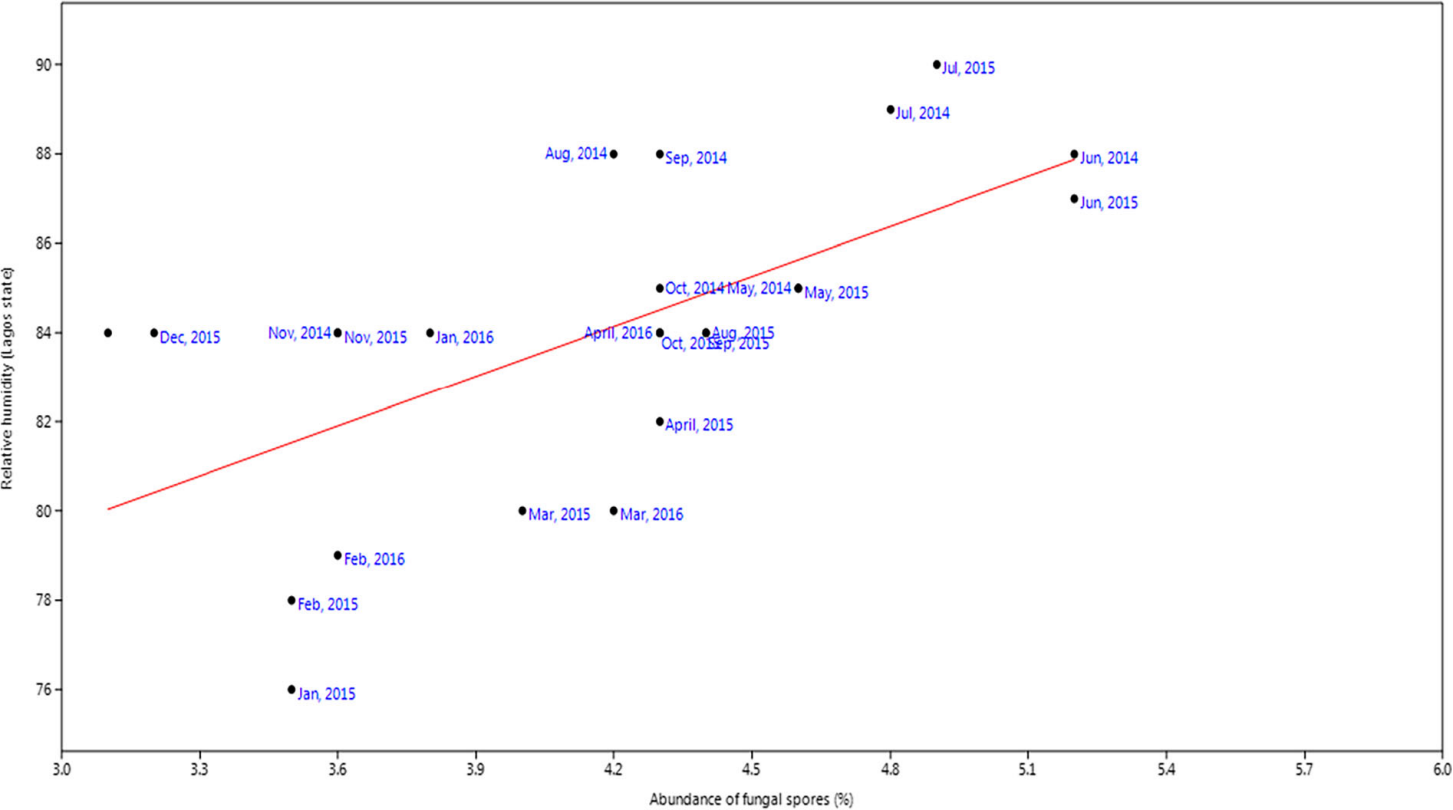
Multivariate linear regression between fungal spore abundance and relative humidity in Lagos

**Fig. 11:**
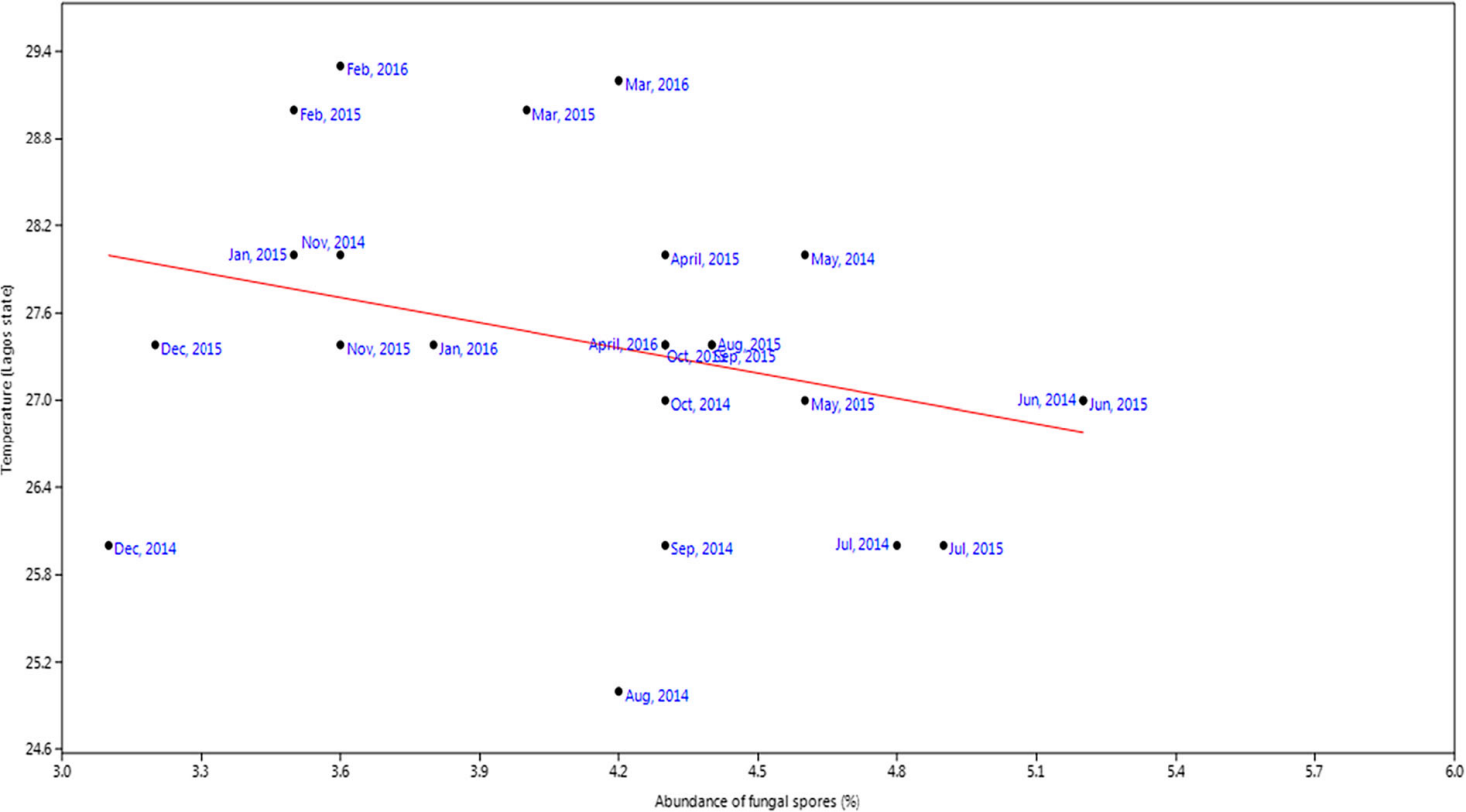
Multivariate linear regression between fungal spore abundance and temperature in Lagos

**Fig. 12:**
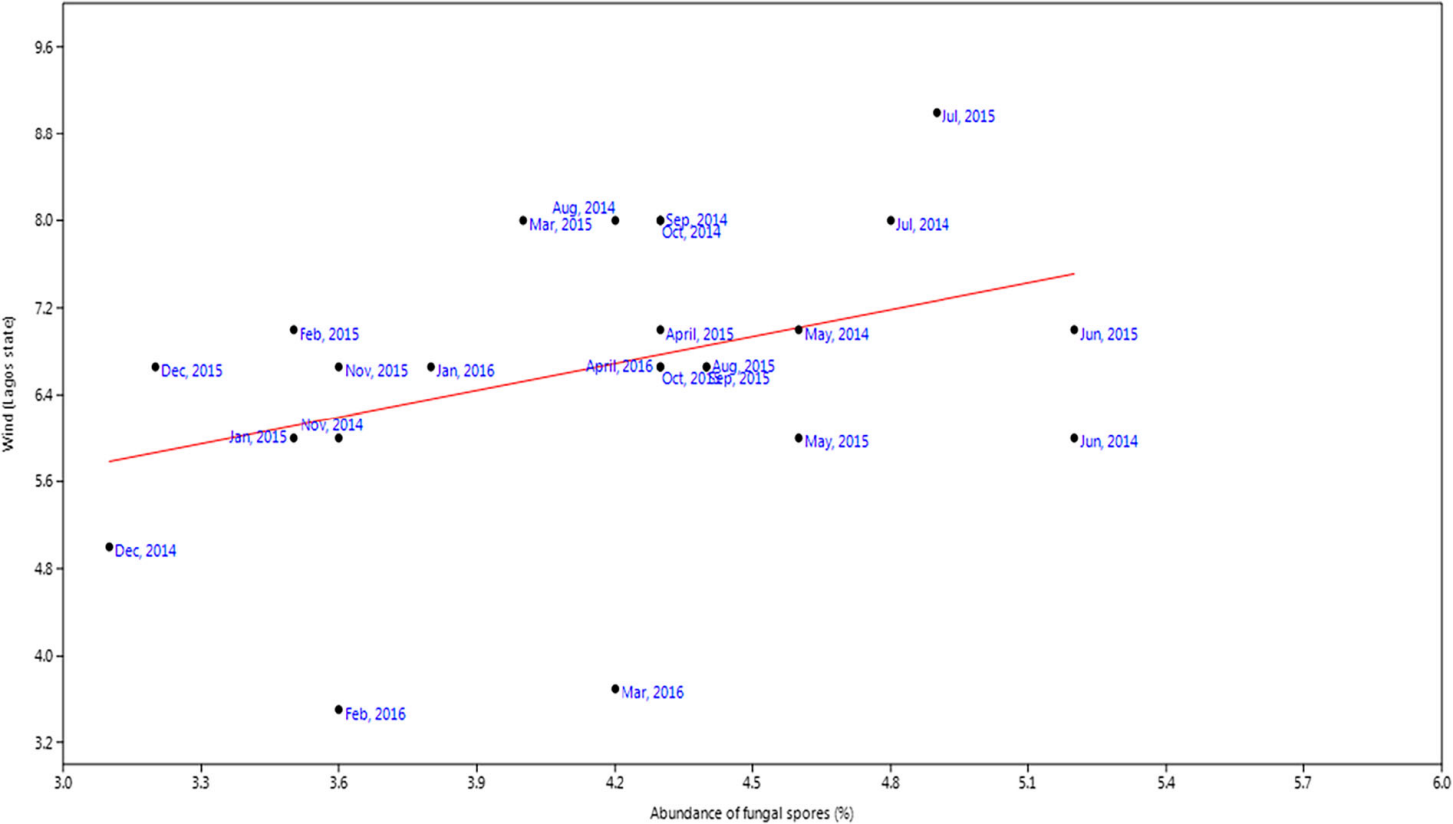
Multivariate linear regression between fungal spore abundance and wind speed in Lagos

For rainfall, the *r* value was 0.20, *P* value was 0.33 while slope was 5.72 (Fig. 9). Relative humidity had slope of 3.74, *P* value of 0.01, and *r* value of 0.62 (Fig. 10). Temperature had slope of − 0.58, *r* value of − 0.31, and *P* value of 0.004 (Fig. 11). For wind, slope was 0.82, *P* value was 0.074, and *r* value was 0.37 (Fig. 12).

**Fig. 13:**
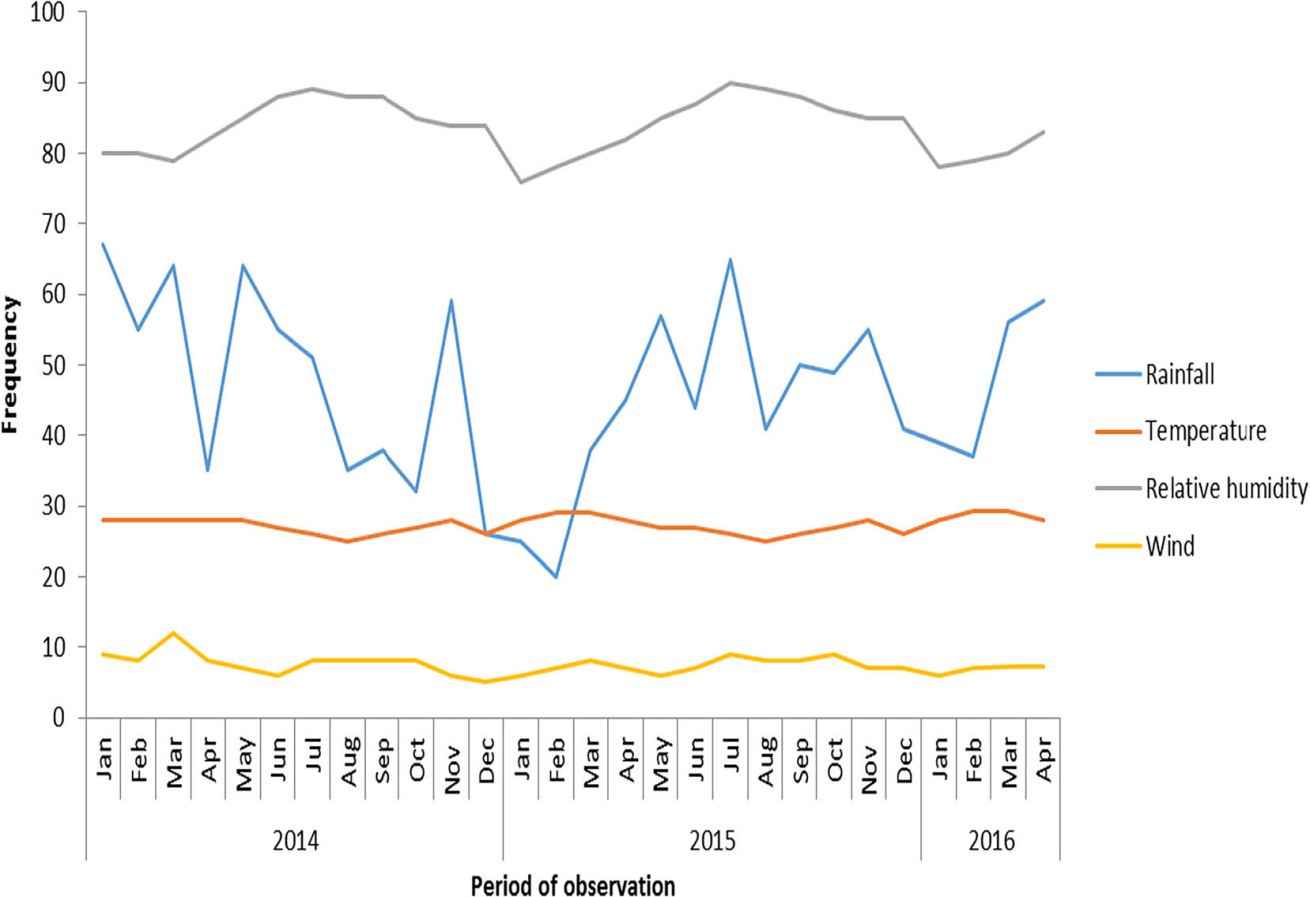
Meteorological data for Lagos State during the period of fungi collection. Source: Nigerian Meteorological Agency

The meteorological data covering the months of May 2014 to April 2016 for Lagos also showed a fairly steady weather parameter for 2 years (Fig. 13).

## Discussion

The occurrence of airborne spores in outdoor environments as well as their abundance, their diversity, and their impact on human, animal, and plant health is gaining attention globally. This study provides information on the relative abundance of fungi from five different locations in Lagos State, Nigeria. The similarities in the distribution and abundance of fungi spores can be influenced by vegetation and close meteorological data (Abdel Hameed et al. 2009). A previous work has examined the composition and dispersal of airborne fungi spores in indoor and outdoor environments in developed countries, but few studies have been carried out in Nigeria. To the best of our knowledge, this is the first study of fungal diversity in the Lagos atmospheric environment, combining identification by direct microscopy with molecular analysis. The combination of the two methodologies allowed us to identify 39 genera and species from airborne spores that make up the environmental mycobiota of Lagos, Nigeria. In this study, fungi isolated included *A*. *flavus*, *Penicillium* spp., *Fusarium* spp., and *Alternaria* spp. which have also been previously found to be abundant airborne species. *Penicillium* spp. and *Aspergillus* spp. were the most frequent fungi that we observed in the cultures. Differences in recovery of fungi on the two media types found that more colonies were typically found on DG-18 agar than PDA in all sampled locations.

There was also significant variation in the abundance of fungi across different times of the year. Fungi were quantitatively high during the rainy (May–October) season and comparatively lesser in the dry (November–March) season, consistent with other observations of fungal abundance periodicity (Rangaswamy et al. 2013). The variations in the airborne fungi at various locations can be said to be as a result of a variety of source environments which include the soils, leaf surfaces, and lush green grasses and various activities going on in the environment. The activities which go on around the environment also play a crucial role in the abundance of these organisms in the atmosphere because it was found out that locations where there was high vehicular and human traffic had more fungal spores in the atmosphere compared with locations like Victoria Island which had low human activities going on in the area. Iba, Ikorodu, and Ikeja recorded the higher number of fungi was likely due to the human activities occurring at these places because they were outdoor market, hospital, and farm settlement areas where hustling and bustling do occur on a daily basis and also high wind speed re-suspends these organisms in the atmosphere. Our work is consistent with a previous report that microbial abundance is correlated with population density and activity (Fang et al. 2007). The hospital environment at Ikeja recorded high fungal spore counts which may be a result of foot and vehicle traffic which increases disturbance. Huang et al. (2014) also observed the same pattern in their work. The reduced number of fungi observed at Victoria Island could be due to prevailing atmospheric conditions since it is an ocean environment and there are few visitors during weekdays.

We found *Aspergillus* species was the most common genus near the hospital location which is consistent with previous findings of fungi from the air in hospital environment (Ekhaise et al., 2008). We also found that *Aspergillus* and *Penicillium* species are the most abundant fungal isolates across the sampled locations. Aerosolized *Aspergillus* spores are found nearly everywhere so humans are routinely and almost constantly exposed to them. These fungal spores contain allergenic protein and it has been reported that exposure to fungal allergens is a strong risk factor for asthma symptom (Denning et al. 2006). Makut et al. (2014) used plate sedimentation method during their investigation of microflora of outdoor air in Nasarawa State, Nigeria, and found that six bacterial species which belong to 6 genera and nine fungal species which belong to 7 genera were identified at various frequencies of distribution. Tsai and Macher (2005) found *A*. *fumigatus* and *A*. *flavus* as the major fungal species in their sampling of the US homes and therefore classified them as the most probable cause of allergic effects and may be possibly hazardous to the health of workers. In our study, a total of thirty-nine different fungal species were identified from various locations. From the *Aspergillus* genus, 12 species were identified of which *A*. *flavus*, *A*. *fumigatus*, *A*. *niger*, and *A*. *terreus* were the most common species sampled while for *Penicillium*, 6 species were identified. Months with high relative humidity and rainfall witnessed significant increase in fungal spore collection throughout the period of sampling. The lowest total number of colonies was obtained in the month of December for both potato dextrose agar and DG-18 agar. The abundance of some of the major fungal spores could be a marker for pathogenicity in the environment and should warn the farmers to protect their crops from diseases. According to Njokuocha (2006), most of the fungi species identified in the air have also been associated with agricultural crop and wild plant diseases in Nsukka, Nigeria. Njokuocha and Ukeje (2006) also proposed that fungal spores are diverse in distribution and represent a large proportion of the airborne spores sampled in most aerobiology studies. Majority of the fungal spores obtained in their work are also among the invasive airborne fungal spores that have been linked with patients with solid organ transplants in hospitals (Sanchez and Bush 2001; Cashel et al. 2004). The internal transcribed spacer (ITS) region has the highest probability of successful identification for the broadest range of fungi. The combinations of both ITS and LSU sequences have been applied in environmental sampling (Gorfer et al. 2011).

## Conclusion

These distribution, frequency, and types of fungi found in this sampling can better inform at risk including those suffering from respiratory diseases and allergy in the types and locations of outdoor activities. It was important to note that fungal spores are ubiquitous but abundance does vary with rainfall and location, and future work to examine the factors that better predict the cycles of fungal spore abundance in Nigeria may provide helpful information to the public and public health agencies.
